# Factors influencing food waste reduction in University Canteens: Toward sustainable campus waste management

**DOI:** 10.1371/journal.pone.0343534

**Published:** 2026-02-23

**Authors:** Patranit Srijuntrapun, Pattama Ket-um

**Affiliations:** 1 Department of Education, Faculty of Social Sciences and Humanities, Mahidol University, Salaya, Thailand; 2 Srinakharinwirot University, Wattana, Thailand; North-Caucasus Federal University, RUSSIAN FEDERATION

## Abstract

**Objective:**

Food waste in university canteens poses a significant challenge to advancing sustainability in higher education. Context-specific, targeted strategies remain limited. This study examines the psychosocial factors influencing food waste reduction behaviors among users of university canteens in Thailand.

**Materials and methods:**

Drawing on ETPB and the waste hierarchy framework, a cross-sectional survey was conducted with 400 undergraduate students in a large Thai university canteen. Data was collected through structured questionnaires and analyzed using descriptive statistics and multiple linear regression to identify key factors influencing food waste reduction behaviors.

**Results:**

While responses showed consistently high levels across all dependent variables, participants engaged moderately in food waste reduction. Multiple regression analysis of the survey’s results only accounted for 17.1% of the variance in food waste reduction behaviors. Perceived behavioral control (β = 0.338, p < 0.001) and motivation (β = 0.162, p = 0.001) emerged as the strongest predictors. The study also found that structural barriers, including poor food quality (65.0%), limited portion size flexibility (36.3%), and time constraints during peak hours (58.8%), hindered upstream food waste prevention.

**Conclusion:**

The findings of this study demonstrate that food waste reduction behavior amongst students is primarily driven by perceived control and motivation rather than knowledge alone. Moreover, although canteens support segregation, upstream prevention is hindered by structural barriers. These findings highlight the need for dual strategies aimed at strengthening psychosocial drivers and improving service environments, in alignment with institutional food waste policies and the global SDG 12.3 targets.

## Introduction

University canteens are underutilized as “strategic spaces” to promote sustainability transitions among the younger generation, particularly regarding food waste reduction. This is a critical but often overlooked component of climate action [1]. Food waste reflects inefficiencies in the use of resources and is a significant contributor to greenhouse gas emissions, exacerbating climate change [2,3] while also contributing to broader environmental degradation [4]. With over 1.3 billion tons of food wasted globally each year [5], there is an urgent need for innovative supply chain measures to reduce waste and promote sustainable consumption behaviors in line with SDG 12.3, which seeks to reduce per capita global food waste by half before 2030 [6].

Research indicates that food waste at the consumption stage has intensified with rising living standards [7]. However, food is lost throughout the supply chain [6] and there are increasing efforts to reduce it, from production to consumption [8–11]. In recent years, there has been growing academic interest in the issue of food waste in university settings [12–14]. Food waste originates in university canteens both from food preparation and consumption [15]. In Thailand, however, canteens do not generally prepare food on-site. In this context, waste is primarily driven by consumer behavior, with students representing a key demographic that contributes significantly to generate food waste [1,16]. Prior research on university food waste has been conducted in Europe, North America, and China, where the focus has often been on awareness campaigns, portion control, and infrastructural measures. However, there remains limited empirical evidence from Southeast Asia. Few studies have systematically tested psychosocial predictors of food waste reduction behaviors in this regional context, despite its distinctive food service systems and cultural practices.

Addressing this gap, the present study investigates the psychosocial determinants of student food waste behaviors in Thai university canteens to inform effective institutional interventions. Unlike most previous studies, which emphasize environmental or operational factors, this work applies multiple linear regression to identify key psychosocial determinants grounded in the Theory of Planned Behavior. The novelty of this research lies in integrating this behavioral framework with the food waste hierarchy, bridging behavioral theory with practical waste management policy. Through this integrative analytical approach, the study provides new empirical evidence to inform institutional strategies and behavioral interventions. The findings of the study offer empirical insights to support policymakers, universities, and researchers in developing effective behavioral interventions and policies aimed at reducing food waste in university canteens across Thailand and in comparable settings.

## Literature review

The Theory of Planned Behavior (TPB) is a widely applied psychological framework for predicting and understanding human behavior across various contexts. According to TPB, behaviors are driven by an individual’s intentions, shaped by three key factors: attitudes toward the behavior (positive or negative evaluations of the behavior), subjective norms (perceived social pressures to engage or not to engage in the behavior), and perceived behavioral control (perceived ease or difficulty in performing the behavior, which is closely related to self-efficacy) [17].

Recent studies support the application of TPB as a robust framework for understanding food waste behaviors in university canteens. In particular, the theory can provide significant predictors of students’ intentions and behaviors toward reducing food waste. It is known that positive attitudes, influenced by environmental awareness, personal values, and perceived benefits, are central in fostering intentions aimed at reducing food waste [13,14,18–20]. Social norms, shaped by peer influence and institutional culture, also encourage waste reduction by creating supportive environments for behavioral change [18,20]. Among these predictors, perceived behavioral control consistently emerges as particularly influential. Research has shown that students who feel capable of managing their portion sizes and food choices are more likely to engage in waste reduction behaviors [1,14,20]. Moreover, interventions like the provision of information, creative campaigns, or portion management have been shown to effectively align attitudes and personal norms with food waste reduction goals [19,21,22].

Recent literature has adopted the Extended Theory of Planned Behavior (ETPB) framework, which retains behavioral intention as a key mediator between psychosocial factors and actual waste reduction behaviors in university canteens [20,23]. This approach incorporates additional variables such as knowledge, motivation, and contextual factors, improving its explanatory power. Knowledge directly influences attitudes and perceived behavioral control, particularly in educational contexts [14,20,21]. Motivation, both intrinsic (e.g., environmental responsibility) and extrinsic (e.g., social recognition), is closely linked to attitudes and intentions to reduce waste [18,23]. Contextual factors, including food quality, canteen environments, and institutional policies, further facilitate or hinder the translation of intention into action [1,13,23]. Challenges such as inadequate distribution systems and limited awareness campaigns add further barriers to sustainable consumption behaviors in higher education settings [15]. Qualitative studies have shown that food waste in university canteens is embedded within complex sociocultural and material contexts, indicating that interventions must address both individual behaviors and systemic structures [12]. Incorporating these variables within the ETPB strengthens its capacity to guide targeted strategies for food waste reduction in university canteens.

The food waste hierarchy is another key framework widely used in the design of waste management policies, particularly those that prioritize strategies to minimize environmental impacts. In this context, prevention is ranked as the preferred option, followed by reuse, recycling, recovery (including energy recovery), and disposal as a last resort [24]. Applied to food waste, the hierarchy prioritizes prevention, for example, through changes in consumer behavior, portion management, or meal planning, over downstream solutions such as composting or anaerobic digestion [25,26].

Most research has tended to align food waste reduction behaviors in university canteens with the food waste hierarchy, emphasizing the priority of prevention. Over-purchasing by students, for example, has been identified as a key driver of food waste, suggesting that appropriate portion sizes and improvements in quality may reduce excessive purchasing [27]. Visschers et al. [19] demonstrated that interventions combining the provision of information with practical behavioral changes could reduce food waste by up to 20%. Digital platforms that remind students to avoid over-purchasing and encourage eating everything on one’s plate can further support waste prevention and facilitate progress tracking [28]. In the case of unavoidable food waste, the recommendation is to separate organic and inorganic waste for recycling or composting [21]. Using the food waste hierarchy as an analytical framework allows researchers and practitioners to assess which consumer behaviors align with waste prevention principles, prioritizing interventions with the greatest environmental benefits. Few studies have explicitly integrated the ETPB and the food waste hierarchy, and this study addresses this gap by combining them to examine both psychosocial intentions and structural enablers of food waste reduction, enabling the design of targeted strategies for effective food waste reduction in university canteens.

## Materials and methods

### Study area

This study was conducted in the canteens of a Thai university. These canteens, which serve over 25,000 undergraduate and graduate students, are operated by various private vendors under university oversight. They play a critical role in providing affordable meals to students and staff. The study site was chosen because it offers a strategic environment where young consumers are developing sustainable consumption and waste segregation behaviors. This aligns with national policy, specifically the Roadmap on Food Waste Management (2023–2030) and the Action Plan on Food Waste Management Phase I (2023–2027), as well as institutional policy goals for waste reduction and environmental sustainability. Most Thai universities have adopted Green University policies and SDG Campus initiatives, which include KPIs for reducing solid and hazardous waste, increasing recycling rates, and improving environmental management on campus [29,30]. However, policies that are specifically targeted at food waste management are not yet well established.

### Data collection and characteristics of participants

A quantitative, cross-sectional survey design was employed to investigate the factors influencing food waste behaviors among canteen customers. Data was collected between May and June 2025 through face-to-face interviews with canteen customers using structured questionnaires. Informants were selected based on eligibility criteria that included being at least 18 years old to ensure informed participation and voluntary consent. As the total population size was unknown, the Cochran [31] formula was used to calculate the required sample size at a 95% confidence level, resulting in a minimum of 385 participants. To enhance statistical reliability, a sample of 400 participants was targeted. A convenience sampling approach was employed due to time and accessibility constraints within the university context. To minimize potential selection bias and ensure the diversity of informants, participants were recruited from a diverse range of faculties and across all major university canteens during different times of the day. Interviews continued until the target sample size was achieved.

### Measurement

The questionnaire used in the interviews was based on the ETPB and the food waste hierarchy approach, incorporating variables previously identified as significant for food waste management in university canteens. The questionnaire was structured into ten sections:

1. General information: Gender, age, and income.
2. Knowledge of food waste: Understanding of food waste issues by participants, assessed using dichotomous (yes/no) items.
3. Attitudes toward food waste reduction: Measured using 5 items rated on a three-point Likert scale (1 = disagree, 2 = neutral, 3 = agree).
4. Motivation for food waste reduction: Evaluated using 4 items rated on a three-point Likert scale (1 = disagree, 2 = neutral, 3 = agree).
5. Perceived behavioral control regarding food waste reduction: Assessed using 3 items on a three-point Likert scale (1 = low, 2 = moderate, 3 = high).
6. Behavioral intention toward food waste reduction: Assessed using 3 items rated on a three-point Likert scale (1 = low, 2 = moderate, 3 = high).
7. Social influence on food waste reduction: Measured using 3 items rated on a three-point Likert scale (1 = low, 2 = moderate, 3 = high).
8. Contextual factors related to food waste reduction: Evaluated using 3 items rated on a three-point Likert scale (1 = low, 2 = moderate, 3 = high).
9. Food waste reduction behavior among customers: Evaluated using 7 items rated on a four-point Likert scale (1 = never, 2 = sometimes, 3 = often, 3 = always).
10. Perceived barriers to food waste reduction: Barriers examined included food waste prevention and food waste segregation (see S1 File).

A three-point Likert scale was adopted to simplify participant responses and minimize ambiguity, ensuring clarity for respondents with diverse educational backgrounds and promoting consistent interpretation across items.

The study used the following independent variables: knowledge, attitudes, motivation, perceived behavioral control, behavioral intention, social influence, and contextual factors, which were selected based on prior evidence of their influence on food waste management behavior. The dependent variable was the behavior of customers aimed at reducing food waste.

To ensure construct validity, all measurement items were adapted from previously validated instruments grounded in ETPB. A theory-driven approach was adopted. Content validity was established through expert review by three specialists in waste management and behavioral science, who assessed item relevance, clarity, and alignment with the ETPB framework. Reliability analysis demonstrated strong internal consistency across all constructs, with Cronbach’s α ranging from 0.721 to 0.845. Because the purpose of this study was to examine the relationships among theoretically predefined constructs and not to develop new measurement scales, the established conceptual structures supported by prior empirical evidence were deemed appropriate for this research context.

### Data analysis

Data was analyzed using SPSS Version 25.0. Descriptive statistics, including frequencies, percentages, means, standard deviations, and ranges, were computed to summarize the demographic characteristics of participants and the key study variables. Multiple linear regression analyses were conducted to examine the relationships among the independent variables (knowledge, attitudes, motivation, perceived behavioral control, behavioral intention, social influence, and contextual factors) and the dependent variable (behavior aimed at reducing food waste among customers in university canteens). Standardized beta coefficients (β), t-values, p-values, 95% confidence intervals, and adjusted R² values were reported to determine the strength and significance of each predictor. All statistical tests were two-tailed, with significance set at p < 0.05.

### Ethical considerations

This study was approved by the Mahidol University Ethics Committee (Certificate of Approval No.2025/020.2003). Prior to data collection, all participants were informed of the study’s objectives, the fact that their participation was voluntary, their right to withdraw at any stage without consequences, and the measures taken to ensure the confidentiality of their personal data. Written informed consent was obtained from all participants, confirming that they were adults (aged 18 years or above). The consent process was formally reviewed and approved by the Ethics Committee to ensure adherence to ethical research standards. All collected data was anonymized and securely stored, with access restricted to the research team for research purposes only.

## Results

### Demographic characteristics

All 400 participants in the study were undergraduate students (100%), who represent the main user group of the university canteens. Most of them were female (n = 319; 79.8%), while male respondents accounted for 20.2% (n = 81). The majority of respondents were under 20 years of age (n = 310; 77.5%), while those aged 21 years and above represented a minority (n = 90; 22.5%). Most participants reported a monthly family income between 15,001 and 30,000 baht (n = 87; 21.75%), closely followed by those with an income of 15,000 baht or less (n = 85; 21.25%). Additional demographic details are given in S1 Table.

### Food waste disposal practices and canteen characteristics

Table 1 summarizes the food waste disposal behaviors of participants alongside the characteristics of the canteens. Most respondents (n = 358; 89.5%) reported disposing of food leftovers directly into designated food waste bins, while a smaller group (n = 42; 10.5%) left them on the plates that were subsequently discarded. Regarding canteen infrastructure, nearly all respondents (n = 382; 95.5%) confirmed the presence of separate food waste bins. An even higher proportion (n = 394; 98.5%) reported the availability of stations where dishes could be dropped off, which indicates that many canteens are well equipped for waste management. However, only 86.0% of respondents (n = 344) reported the presence of clear signage promoting food waste separation, suggesting opportunities for improvement in behavioral prompting.

**Table 1:** Food Waste Disposal Behaviors and Canteen Characteristics.

Item	n	%
**Food waste disposal behaviors in the canteen**		
Dispose of food leftovers directly into designated food waste bins	358	89.50
Place plates with leftover food in the dish collection area	42	10.50
**Canteen characteristics**		
Designated food waste bins are available	382*	95.50
Dish, bowl, and utensil collection points are available	394*	98.50
Signage promoting food waste separation is present	344*	86.00
**Vendor characteristics**		
Clear signage indicates the availability of various portion sizes	220*	55.00
No signage, but customers can request reduced portions	344*	86.00
Bags or containers to take away excess food are available	245*	61.40

*The survey allowed respondents to choose more than one option.

Concerning portion sizes, 55.0% of respondents (n = 220) reported that vendors displayed clear indications of sizes, while 86.0% of them (n = 344) noted the absence of such indications but reported that customers could specify portion sizes upon ordering. Additionally, 61.4% of respondents (n = 245) reported that canteens provided packaging for leftover food to be taken away.

Overall, these findings highlight that there is substantial infrastructural support for waste separation in the university canteens. However, gaps remain in upstream prevention efforts, particularly in promoting portion size management and encouraging vendor practices that enable customers to reduce food waste at the point of purchase.

### Independent variables

The descriptive statistical analysis of the psychological factors related to food waste behavior among university canteen customers (n = 400) showed consistently high levels across all measured variables (Table 2). Specifically, **knowledge** of food waste reduction was high (M = 5.34, SD = 0.802, α = 0.721), with 99.75% of respondents acknowledging that purchasing appropriate quantities of food could reduce waste and 98.50% recognizing the role that food scraps used as animal feed could play in minimizing canteen waste (see S2 File). **Attitudes** toward food waste reduction in the university canteens were also positive (M = 2.81, SD = 0.281, α = 0.770), with 95.50% of respondents agreeing that everyone should take responsibility for their own food waste and 81.75% expressing the opinion that waste sorting was not burdensome (see S2 File). These results seem to provide a constructive foundation for positive behavioral change. Moreover, among all factors, the **motivation** to reduce food waste achieved the highest mean score (M = 2.88, SD = 0.218, α = 0.787), with 95.75% of respondents saying that they were willing to comply with clear canteen regulations and designated sorting points, while 90.25% reported that they already separated food waste as a way to contribute to reduce pollution and conserve the environment (see S2 File).

**Table 2:** Summary of Independent Variables.

Factor	Mean	S.D.	Min	Max	Reliability (Cronbach’s α)
Knowledge**	5.34	0.802	2.00	6.00	0.721
Attitude*	2.81	0.281	2.67	2.95	0.770
Motivation*	2.88	0.218	2.86	2.96	0.787
Perceived behavioral control*	2.63	0.367	2.60	2.71	0.765
Behavioral intention*	2.85	0.275	2.81	2.90	0.806
Social influence*	2.73	0.375	2.62	2.83	0.845
Contextual factors*	2.84	0.305	2.72	2.92	0.783

*Mean scores were categorized into three levels: low (1.00–1.66), moderate (1.67–2.33), and high (2.34–3.00).

**Mean scores were categorized into three levels: low (1.00–2.99), moderate (3.00–4.99), and high (5.00–6.00).

In addition, **perceived behavioral control** regarding food waste reduction amongst respondents was relatively high (M = 2.63, SD = 0.367, α = 0.765), even if there was significant variability, with 60.50% expressing confidence in their ability to reduce food waste and 70.50% in correctly sorting food waste (see S2 File). Respondents also showed a strong **intention to modify their behavior** in order to reduce food waste (M = 2.85, SD = 0.275, α = 0.806), with 90.50% of them planning to correctly sort it in the designated bins and 84.50% indicating that they would purchase food portions adjusted to their consumption (see S2 File). Responses also showed a strong influence of **social influence** on food waste reduction (M = 2.73, SD = 0.375, α = 0.845), with 82.80% of participants expressing the belief that the university played a role in promoting waste reduction behaviors and 78.00% indicating that sorting food waste was a reflection of social responsibility (see S2 File). Finally, contextual factors related to food waste reduction achieved high scores (M = 2.84, SD = 0.305, α = 0.783), with 92.75% of respondents indicating that the presence of separate bins facilitated the sorting process and 88.50% noting that directional signage also supported these behaviors (see S2 File). Overall, the various instruments used in the study were internally consistent, with Cronbach’s alpha coefficients ranging between 0.721 and 0.845 across all variables.

### Dependent variable

This study examined behaviors aimed at reducing food waste among canteen customers using the food waste hierarchy framework, with survey items tailored to this particular context. Five key behaviors were assessed: purchasing only necessary amounts to prevent waste, consuming appropriate portions and sharing food, reusing food through redistribution, utilizing food waste for other beneficial purposes, and disposing of food waste in landfills. Overall, respondents demonstrated a moderate level of food waste reduction behaviors (M = 2.98) (Table 3).

**Table 3:** Summary of Food Waste Reduction Behaviors Among University Canteen Customers by Frequency (n = 400).

Behaviors	Always	Often	Sometimes	Never	($\bar{x}\;$)	Interpretation
**Purchasing only necessary amounts to prevent waste**						
1. Purchasing only amounts of food that can be completely consumed.	165 (41.25)	158 (39.50)	73 (18.25)	4 (1.00)	3.21	High
2. Requesting food vendors to exclude items that are not eaten, such as certain vegetables, or adjusting rice portions based on preference.	144 (36.00)	107 (26.75)	116 (29.00)	33 (8.25)	2.91	Moderate
**Subtotal**					**3.06**	**High**
**Consuming appropriate portions and sharing food**						
3. Finishing all the food ordered in the canteen.	178 (44.50)	152 (38.00)	70 (17.50)	0 (0.00)	3.27	High
4. Leaving uneaten food on the plate when disliked.	79 (19.75)	99 (24.75)	191 (47.75)	31 (7.75)	2.57	Moderate
**Subtotal**					**2.92**	**Moderate**
**Reusing food**						
5. Taking surplus food home after the meal.	27 (6.75)	56 (14.00)	183 (45.75)	134 (33.50)	1.94	Low
**Subtotal**					**1.94**	**Low**
**Utilizing food waste for other beneficial purposes**						
6. Separating tissue or other waste from food to support its reuse, for example as animal feed.	258 (64.50)	88 (22.00)	43 (10.75)	11 (2.75)	3.48	High
**Subtotal**					**3.48**	**High**
**Disposal in landfills**						
7. Disposing of excess food in designated bins to reduce the amount of waste sent to landfills.	263 (65.75)	82 (20.50)	51 (12.75)	4 (1.00)	3.51	High
**Subtotal**					**3.51**	**High**
**Total**					**2.98**	**Moderate**

Based on the analysis of the behaviors shown in Table 3, respondents seemed to use waste reduction strategies at the disposal stages more frequently than preventive strategies. For example, “disposing of excess food in designated bins to reduce the amount of waste sent to landfills” received the highest mean score (M = 3.51), with 65.75% of participants reporting that they “always” engaged in this behavior. This seems to indicate a clear commitment to reducing landfill waste. Similarly, “separating tissue or other waste from food to support its reuse, for example as animal feed” also received a high mean score (M = 3.48), reflecting behaviors that facilitate the safe reuse of food waste.

In contrast, preventive behaviors were less frequent. For instance, while “purchasing only amounts of food that can be completely consumed” achieved a high mean score (M = 3.21), “requesting food vendors to exclude items that are not eaten, such as certain vegetables, or adjusting rice portions based on preference” only had a moderate mean score (M = 2.91). Notably, 29.00% of participants reported that they “sometimes” practiced this behavior, while 8.25% responded that they “never” did. This suggests that downstream waste management is more normalized, reflecting infrastructural readiness but diverging from the waste hierarchy’s emphasis on prevention. This highlights gaps in preventive behaviors that could be addressed through targeted interventions and food waste reduction initiatives at the university.

Categorizing these behavioral scores into three levels, 83.5% of respondents fell within the high range (scores 15–21), while 16.25% were in the moderate range (scores 8–14), and 0.25% in the low range (scores 0–7). These findings point to opportunities to strengthen upstream prevention behaviors that may complement existing efforts to reduce and reuse waste, advancing the university’s sustainability goals.

### Factors Influencing Food Waste Reduction in the University Canteens

To identify the factors influencing behaviors aimed at reducing food waste in the university canteens, the study first tested the preliminary assumptions required for multiple regression analysis, such as normality of distribution, linearity, and independence of residuals, all of which met the criteria for appropriate analysis. To further ensure the robustness of the regression model, multicollinearity diagnostics were conducted. Tolerance and Variance Inflation Factor (VIF) values were examined. Tolerance values exceeded 0.10 (ranging from 0.55 to 0.81), while all VIF values were below 5 (ranging from 1.17 to 1.81). These values fall within the acceptable thresholds suggested by Hair et al. [32]. In addition, all Condition Index (CI) values were below 30, with the highest value being 25.00. These findings confirm that the regression model is not affected by multicollinearity, which could otherwise distort the estimation of regression coefficients.

Subsequently, Pearson’s correlation analysis, summarized in Table 4, revealed that most independent variables were positively correlated with food waste reduction behaviors at a 0.01 significance level. In particular, motivation (r = . 256), perceived behavioral control (r = .383), and behavioral intention (r = .321) showed significant positive correlations, while attitude exhibited a weaker degree of positive correlation (r = .135). Finally, it was found that knowledge about food waste reduction had a weak negative correlation with the behaviors of interest (r = −.050).

**Table 4:** Analysis of the relationships between variables.

Variables	1	2	3	4	5	6	7	8
Knowledge (1)	–	.384**	.240**	−.028	.133**	.160**	.106**	−.050
Attitude (2)		–	.340**	.213**	.316**	.328**	.052	.135**
Motivation (3)			–	.277**	.457**	.379**	.271**	.256**
Perceived behavioral control (4)				–	.527**	.264**	.201**	.383**
Behavioral intention (5)					–	.468**	.247**	.321**
Social influence (6)						–	.300**	.227**
Contextual factors (7)							–	.190**
Behaviors (8)								–

** Significance level of 0.01

The correlation results reveal the interplay between psychological and contextual determinants in shaping food waste reduction behaviors. The strongest relationship was observed between perceived behavioral control and behavioral intention (r = .527, p < .01). This suggests that, when individuals feel confident in their ability to manage or reduce food waste, they are more likely to develop stronger intentions to act accordingly. This finding aligns with ETPB, which posits that perceived control increases both behavioral intention and the probability of action.

Motivation also appeared to be a critical driver, showing moderate to strong correlations with behavioral intention (r = .457) and social influence (r = .379). This indicates that students are much more likely to commit to sustainable consumption when they perceive strong social support and positive norms. Positive messages from peers, teachers, or the campus community can help students feel that their actions matter and that they are part of a collective effort.

In addition, contextual factors showed a weaker but still significant relationship with behavior (r = .190). This finding highlights how providing environmental support, such as accessible bins, clear signage, and effective canteen waste management systems, can encourage people to dispose of waste properly. These relationships correspond with the behavioral patterns observed in Table 3, where downstream actions such as waste separation and appropriate disposal were more prevalent than upstream prevention behaviors.

However, it was also found that knowledge has a negative and weak correlation with actual behavior (r = −.050). This means that information alone may not be sufficient to drive behavioral change without the reinforcement of motivation and contextual support. Overall, the results suggest that the moderate level of food waste reduction behavior by students (M = 2.98) is largely influenced by their perceived capability, motivation, and structural support within the canteen environment, rather than by knowledge or attitudes alone. This finding underscores the importance of integrated strategies aimed at strengthening both personal agency and environmental facilitation to foster sustainable and preventive food waste practices in university contexts.

To further identify and investigate the predictive factors, a multiple regression analysis using the Enter method was conducted. As shown in Table 5, the model accounted for 17.1% of the variance in food waste reduction behaviors (R² = .171, p < .001). Significant predictors included perceived behavioral control (β = .265, p < .001), motivation (β = .117, p = .032), and knowledge (β = −.111, p = .027). On the other hand, attitude, behavioral intention, social factors, and environmental factors did not appear to have a significant influence.

**Table 5:** Multiple regression coefficients for predictors of food waste reduction behaviors in the university canteens (First Analysis).

Variables	b	SE	Beta	t	p-value
Constant	0.953	0.293		3.250**	0.001
Knowledge	−0.056	0.025	−0.111	−2.218*	0.027
Attitude	0.045	0.076	0.032	.597	0.551
Motivation	0.214	0.100	0.117	2.153*	0.032
Perceived behavioral control	0.289	0.059	0.265	−4.877***	0.000
Behavioral intention	0.126	0.089	0.087	1.424	0.155
Social influence	0.064	0.061	0.057	1.040	0.297
Contextual factors	0.101	0.064	0.077	1.507	0.885
R = 0.414, R^2^ = 0.171, F = 41.002					

Note: *p < 0.05, **p < 0.01, ***p < 0.001. Enter multiple regression analysis.

To assess the stability of the identified predictors, an additional regression analysis was performed. This analysis retained only the most significant variables (knowledge, perceived behavioral control, motivation). As shown in Table 6, this model explained the same proportion of variance (R² = .171) but only perceived behavioral control (β = .338, p < .001) and motivation (β = .162, p = .001) remained statistically significant, while knowledge had to be dropped. From a behavioral science perspective, these findings suggest that perceived behavioral control and motivation are the primary predictors of food waste reduction behaviors, whereas knowledge is a weaker predictor when controlling for other variables.

**Table 6:** Multiple regression coefficients for predictors of food waste reduction behaviors in the university canteens (Second Analysis).

Variable	b	SE	Beta	t	p-value
Constant	1.150	0.252		4.567***	<0.001
Perceived behavioral control	0.369	0.052	0.338	7.110***	<0.001
Motivation	0.298	0.087	0.162	3.416***	0.001
R = 0.414, R^2^ = 0.171, F = 41.002					

Note: ***p < 0.001. Enter multiple regression analysis.

The raw score form of the predictive equation for food waste reduction behaviors in the university canteens is as follows:

Food Waste Reduction Behavior = 1.150 + 0.369 * (Perceived behavioral control) + 0.298 * (Motivation)

When converted to standardized Z-scores, the predictive equation becomes:

Food Waste Reduction Behavior = 0.338 * (Perceived behavioral control) + 0.162 * (Motivation)

This equation can guide the development of interventions in university settings, emphasizing the need to enhance the perceived behavioral control and motivation of customers in order to foster sustainable food waste reduction behaviors.

### Perceived barriers to food waste reduction

The survey identified several barriers to food waste reduction. As shown in Table 7, the primary barrier preventing respondents from reducing their food waste was the perceived poor taste or low quality of the food served in the university canteens (65.00%), which constituted the main reason for leftovers. This was followed by a lack of knowledge about appropriate portion planning (38.80%) and the inability to select portion sizes according to individual needs (36.30%).

**Table 7:** Perceived barriers to food waste reduction.

Barrier	*n* (%)
The food served does not taste good or is of low quality.	260 (65.00)
Lack of knowledge on how to appropriately plan the quantity of food to purchase.	155 (38.80)
No options available for selecting portion sizes according to individual needs.	145 (36.30)
Reluctance to request food vendors to reduce the amount of rice or food served.	139 (34.80)
Insufficient information from the canteen regarding food waste reduction initiatives.	78 (19.50)
Lack of motivation to reduce food waste.	71 (17.80)
Other reasons, such as perceiving no benefits in reducing food waste.	14 (3.50)

*Note: The survey allowed respondents to choose more than one option.*

In addition, as shown in Table 8, the survey revealed that the primary barrier to food waste segregation was the existence of time constraints during peak service hours (58.80%). This was followed by a lack of knowledge regarding proper segregation behaviors (47.30%) and the absence of dedicated food waste bins in the canteens (22.30%). These findings suggest that psychosocial drivers alone may be insufficient unless structural barriers are simultaneously addressed.

**Table 8:** Perceived barriers to food waste segregation.

Barrier	*n* (%)
Time constraints during peak hours.	235 (58.80)
Lack of awareness regarding proper methods for separating food waste.	189 (47.30)
Absence of designated food waste bins in the canteen.	89 (22.30)
Inconvenient access to food waste bins.	63 (15.80)
Perception that failing to dispose of food waste in designated bins carried no personal consequences.	62 (15.50)
Unwillingness to separate food waste from plates due to its unpleasant or dirty nature.	46 (11.50)
Other reasons, such as reluctance to handle waste.	12 (3.00)

Note: The survey allowed respondents to choose more than one option.

## Discussion and conclusions

Using multivariate linear regression analysis, this study has identified perceived behavioral control (β = .338) and motivation (β = .162) as the primary predictors of food waste reduction behaviors in university canteens. Although the model explains 17.1% of the variance in food waste reduction behavior, this level is consistent with prior research employing TPB and its extensions. Behavioral outcomes in waste management are inherently complex and influenced by a wide range of individual, social, and contextual factors. They were not fully captured within the present model. Such levels of explanatory power are commonly reported in studies examining multifaceted pro-environmental behaviors [33,34]. The results of the study show that perceived behavioral control (β = .338) is a key driver of food waste reduction. This finding aligns with TPB, which emphasizes the role of internal factors, particularly perceived behavioral control [17,35]. Previous studies [1,14,20,36] have reached similar conclusions, showing that confidence in one’s ability to control food waste behaviors is significantly associated with actual practice.

These results support the view that students’ perception of their own control over their eating behaviors in the canteen decreases the food waste they tend to generate [1,14,20,36]. This is because food waste reduction behaviors, such as avoiding over-purchasing, finishing meals, or properly segregating waste, are individual actions that can be immediately enacted under personal control rather than relying on shared social norms. As they are often exposed to environmental education and ongoing waste segregation campaigns, university students tend to believe that they can manage their consumption behaviors and are willing to act directly to reduce food waste [14,37,38].

The study has also revealed that 70.5% of respondents segregated food waste with high confidence, whereas 60.5% felt confident in reducing food waste while using canteen services (S2 File). This discrepancy indicates that respondents are more confident in “waste segregation” than they are in “waste prevention.” In addition, 95.5% of respondents reported the availability of designated food waste bins, but only 55.0% found clear signage on portion size options (Table 1). This indicates that, while “segregation infrastructure” is well-established, limitations in portion flexibility remain a barrier to “prevention efforts.”

These findings highlight that upstream waste prevention, prioritized within the food waste hierarchy, continues to face structural challenges in university canteens, while downstream segregation for recycling is more readily supported and practiced.

Thus, it can be inferred that the availability and accessibility of supportive infrastructure plays a crucial role in strengthening students’ perceived behavioral control (PBC). This aligns with the findings of Waxin et al. [39], who reported a positive association between PBC and convenience infrastructure. Their study suggests that students’ confidence in managing food waste tends to increase when they perceive the canteen environment as being equipped with adequate and user-friendly facilities. Moreover, Visschers [19] further highlighted that convenient structural interventions, such as providing smaller portion sizes together with appropriate informational cues, can effectively enhance the individuals’ perceived control over food waste reduction behaviors by individuals.

Motivation (β = .162) was found to be another key driver of food waste reduction behavior and consumer action. This aligns with ETPB, which integrates motivation and other psychosocial factors to enhance predictive accuracy. For example, Chen [23] demonstrated that motivation explained 56.84% of the variance in the intention to avoid food waste in Taiwan, while Aktas et al. [18] identified motivation in food choices as a contextual factor that predicted food waste behaviors beyond traditional TPB variables. Motivation not only influences intentions but also directs behavior, offering a valuable entry point for designing policies and interventions aimed at encouraging food waste reduction.

The study has also identified several barriers to food waste prevention (Table 7), such as poor food quality or unappealing taste (65.0%), lack of knowledge about portion planning (38.8%), and the lack of flexibility in portion size choice (36.3%). Regarding food waste segregation (Table 8), barriers included time constraints during peak hours (58.8%), limited awareness of proper segregation behaviors (47.3%), and the absence of dedicated food waste bins (22.3%).

These constraints may help explain why respondents demonstrated relatively high overall knowledge scores, but only their actual food waste reduction behaviors remained moderate. The study also found a weak negative correlation between knowledge and behavior (r = −.050). This discrepancy reflects the well-known knowledge-behavior gap, which is a phenomenon widely documented in sustainability and environmental psychology research [40,41]. Although students may be aware of appropriate strategies for reducing food waste, they still struggle to translate this knowledge into practice due to limited motivation or inadequate canteen infrastructure. Examples include the lack of accessible food waste bins, the absence of portion size controls, and restricted mealtimes, all of which may hinder their ability to convert knowledge into action [19,42]. Furthermore, awareness generated solely through campaigns or classroom-based instruction may be insufficient to foster sustained behavioral commitment without experiential engagement or supportive environmental structures [43].

In conclusion, this study examined the psychosocial factors influencing food waste reduction behaviors among university canteen customers in Thailand, using ETPB and the food waste hierarchy framework through multivariate regression analysis. The key findings indicate that perceived behavioral control (β = .338) and motivation (β = .162) are the primary drivers of food waste reduction behavior, highlighting that students who feel confident in their ability to manage food waste and who are motivated are more likely to engage in waste reduction practices.

Despite 95.5% of respondents reporting the availability of designated food waste bins, only 55.0% found clear signage on portion size options and only 60.5% felt confident in their ability to reduce food waste while using the canteen. Grounded in empirical evidence, these findings support a dual approach. The interventions should address both individual-level factors (motivation and perceived control) and environmental or operational factors (infrastructure and flexible portion sizing). The explicit links between β values, respondent confidence percentages, and identified barriers provide empirical support for this conclusion.

Building on these results, a systemic, multi-level approach is recommended for sustainable food waste management in university canteens [44]. This approach integrates behavioral insights with coordinated action across multiple levels of the university ecosystem. This includes policy, organizational structures, operational strategies, and stakeholder engagement. To embed food waste reduction within a multi-level system, universities can foster sustainable consumption behaviors and enhance student motivation and perceived control, while contributing to achieving national food waste reduction goals.

### Research implications

***Theoretical Implications:*** This study improves the theoretical understanding of food waste reduction efforts in university canteens by identifying perceived behavioral control and motivation as primary predictors of these behaviors. These results reinforce a central tenet of TPB, particularly regarding the role of perceived behavioral control in translating intention into action [17]. At the same time, the significance of motivation, a construct outside the original TPB, suggests that extending TPB to include motivational factors offers greater explanatory power for predicting food waste reduction behaviors. While TPB remains a robust framework for explaining pro-environmental actions, the complexity of food waste reduction in service systems and local operational contexts suggests the need to expand TPB to include motivation as a psychosocial factor that can improve behavioral prediction. These insights support the development of a model focusing on variables with demonstrated behavioral and systemic influence, guiding future research, intervention design, and the testing of ETPB models across diverse contexts.

***Policy Implications:*** The findings of this study provide empirical support for policies that integrate improvements in service infrastructure with initiatives that enhance consumer motivation and perceived behavioral control to reduce and segregate food waste. This approach aligns with Thailand’s Roadmap on Food Waste Management (2023–2030) and Action Plan on Food Waste Management Phase I (2023–2027), which emphasize prevention and reduction of food waste at the source while developing efficient localized management systems. A critical insight is that while segregation behaviors are better established, upstream prevention remains underdeveloped, even though it is prioritized in the food waste hierarchy. This reflects structural barriers such as food quality and portion inflexibility, indicating that behavioral interventions must be paired with systemic changes in food service practices.

As key institutional stakeholders, universities should implement effective policies in their canteens, for example by offering flexible portion sizes to prevent food waste, taking measures to facilitate organic waste segregation, and establishing food waste monitoring systems that help refine ongoing policies. These initiatives should be integrated into the green university policies and sustainable development plans as a way to foster collaboration towards the goal of systematically reducing and segregating food waste among students, food vendors, and university staff. Such measures can help universities achieve the SDGs in concrete and effective ways.

***Practical implications:*** The findings of this study provide practical strategies for promoting food waste reduction and segregation in university canteens through a systemic, multi-level approach. This approach helps address identified barriers and fosters sustainable behavioral change. **At the individual level**, students can engage in experiential learning activities such as tracking weekly food waste, calculating associated carbon footprints, and using digital platforms to monitor behavior. These activities enhance awareness, perceived behavioral control, and motivation [43]. Motivational campaigns can further strengthen psychosocial drivers for sustained behavioral change [18,23].

**At the operational and canteen level**, specific methods to induce behavioral change include flexible portion sizing and production planning based on actual consumption data to reduce waste at the source, clear labelling and accessibility of organic waste bins, and informative signage to guide proper segregation [19,21]. Active engagement of canteen staff is essential to support behavioral changes. It also enables staff to interact with consumers and implement ongoing campaigns, including measures to reduce peak-hour bottlenecks [12,19]. **At the organizational and policy level**, establishing dedicated sustainability committees and integrating food waste prevention into university policies requires clear targets, accountability mechanisms, and coordinated actions across units. These efforts not only reduce food waste but also help strengthen a university-wide culture of sustainability [45,46]. Continuous monitoring and reporting, such as documenting reductions in food waste volumes or tracking composting outcomes, reinforces motivation and perceived control [42].

**At the stakeholder and community level**, the engagement of students, food vendors, and surrounding communities promotes shared responsibility. It also encourages initiatives such as redirecting food waste for composting by local farmers or implementing hands-on, systems-thinking experiential programs [42]. Together, these strategies emphasize a dual focus: enhancing psychosocial drivers while addressing structural and operational barriers. In this way, food waste reduction can be embedded within a multi-level university ecosystem. This can support sustainable consumption behaviors aligned with national food waste management targets [19,23,40].

## Limitations and future research

This study has some limitations that should be interpreted within the context of behavioral research in university settings. First, the data were collected through self-administered questionnaires. This approach may introduce a degree of self-report bias, which is a common challenge in studies measuring environmentally related behaviors. Additionally, the study relied on a convenience sample of 400 undergraduate students. In the context of a university canteen, this represents the most accessible population. Such sampling may limit the findings’ representativeness and possibly introduce selection bias. Therefore, the results should be interpreted with reasonable caution when generalizing to other user groups such as staff or visitors. The use of a three-point Likert scale is another methodological consideration. This simplified format enhanced clarity and respondent efficiency in the fast-paced canteen environment. However, it may have reduced the variability of attitudinal responses.

Future research could address these considerations by employing probability-based sampling methods. More diverse user groups would also help enhance representativeness across the university community. Comparative analyses among students, staff, and external visitors may also offer a more nuanced perspective on food waste-related behaviors, for example, by revealing how each group’s distinct routines may shape their disposal choices in different ways. Additionally, adopting more granular Likert scales in future studies may help capture a wider range of attitudinal variation. This approach could deliver more substantive findings without compromising clarity or respondent engagement. Longitudinal research designs could further strengthen the evidence base. They offer insights into the development of such behaviors over time, including how students gradually internalize waste-reduction habits as they encounter repeated prompts or campus-wide campaigns.

## Supporting information

S1 File Original survey questionnaire used in the study. (DOCX)

S1 Table Demographic characteristics. (DOCX)

S2 File Result of independent variables. (DOCX)
