# Supplementary material for: Factors influencing food waste reduction in University Canteens: Toward sustainable campus waste management
Source: PLoS One. 2026 Feb 23;21(2):e0343534. doi: 10.1371/journal.pone.0343534 (PMC12928407; doi:10.1371/journal.pone.0343534)
Supplement: S1 File — (DOCX) [file pone.0343534.s001.docx]

**S1 File. Original survey questionnaire used in the study**

**Section 1** Demographic characteristics

Gender ❒ Male ❒ Female ❒ Other

Age ……

Monthly income ❒ Less than 15,000 baht (< $424) ❒ 15,001 – 30,000 baht ($424-$848) ❒ 30,001 – 45,000 baht ($849-$1,272) ❒ 45,001 – 60,000 baht ($1,273-$1,697) ❒ 60,001 – 75,000 baht ($1,698-$2,121) ❒ More than 75,000 baht (> $2,121)

**Section 2** Knowledge of food waste reduction

| **Knowledge question** | **True (*n*, %)** | **False (*n*, %)** |
| --- | --- | --- |
| 1. Purchasing food served by vendors without prior planning, leading to leftover waste |  |  |
| 2. Leaving food unfinished or uneaten, contributing to global warming |  |  |
| 3. Polluting wastewater from food decomposition does not cause soil and water pollution |  |  |
| 4. Purchasing appropriate quantities of food could reduce waste |  |  |
| 5. Recognizing the role that food scraps used as animal feed could play in minimizing canteen waste |  |  |
| 6. Feeding animals with food scraps can causes minimizing food waste canteen |  |  |

**Section 3 Attitudes toward food waste reduction in the university canteens**

| **Attitude** | Agree | Not sure | Disagree |
| --- | --- | --- | --- |
| 1. Everyone should take responsibility for their own food waste. |  |  |  |
| 2. Leftover or uneaten food decomposes easily and does not harm the environment. |  |  |  |
| 3. It is unnecessary for people in society to be aware of food waste impacts because other environmental problems are more serious. |  |  |  |
| 4. Sorting food waste in canteens is complicated and time-consuming. |  |  |  |
| 5. Sorting food waste in canteens should be the responsibility of canteen staff only. |  |  |  |

**Section 4 Motivation to reduce food waste in the university canteens**

| **Motivation** | **Agree** | **Not sure** | **Disagree** |
| --- | --- | --- | --- |
| 1. I purchase an appropriate amount of food in the canteen, avoiding excess, to save unnecessary food expenses. |  |  |  |
| 2. I reduce food waste in the canteen because I feel it helps conserve the resources used to produce food globally. |  |  |  |
| 3. I separate food waste into designated bins because I feel it helps reduce pollution and protect the environment. |  |  |  |
| 4. I am willing to follow clear canteen rules and use designated bins for food waste separation sincerely if such regulations and facilities are provided. |  |  |  |

**Section 5 Perceived behavioral control regarding food waste reduction**

| **Perceived behavioral control** | **High** | **Moderate** | **Low** |
| --- | --- | --- | --- |
| 1. I am confident that I can reduce food waste when using the canteen. |  |  |  |
| 2. I can order food in quantities that match my consumption needs. |  |  |  |
| 3. I am confident that I can properly separate food waste into designated bins. |  |  |  |

**Section 6 Behavioral intention in order to reduce food waste**

| **Behavioral intention** | **High** | **Moderate** | **Low** |
| --- | --- | --- | --- |
| 1. I intend to reduce food waste when using the canteen. |  |  |  |
| 2. I intend to order food in quantities that match my consumption needs. |  |  |  |
| 3. I intend to separate food waste into designated bins properly. |  |  |  |

**Section 7 Social influence on food waste reduction**

| **Social influence** | **High** | **Moderate** | **Low** |
| --- | --- | --- | --- |
| 1. Society views separating food waste into designated bins as a socially responsible behavior. |  |  |  |
| 2. People around me, including friends and colleagues, support and encourage me to reduce food waste in the canteen. |  |  |  |
| 3. The university’s efforts to promote food waste reduction support customers’ food waste reduction behaviors |  |  |  |

**Section 8 Contextual factors related to food waste reduction**

| **Contextual factors** | **High** | **Moderate** | **Low** |
| --- | --- | --- | --- |
| 1. Having signs that indicate appropriate food portions helps reduce food waste. |  |  |  |
| 2.Having designated bins for food waste helps facilitate food waste separation. |  |  |  |
| 3. Having directional signs to food waste separation points supports food waste separation. |  |  |  |

**Table 9 Summary of** **Food Waste Reduction Behaviors Among University Canteen Customers by Frequency**

| **Behaviors** | **Always** | **Often** | **Sometimes** | **Never** |
| --- | --- | --- | --- | --- |
| 1. Purchasing only amounts of food that can be completely consumed. |  |  |  |  |
| 2. Requesting food vendors to exclude items that are not eaten, such as certain vegetables, or adjusting rice portions based on preference. |  |  |  |  |
| 3. Finishing all the food ordered in the canteen. |  |  |  |  |
| 4. Leaving uneaten food on the plate when disliked. |  |  |  |  |
| 5. Taking surplus food home after the meal. |  |  |  |  |
| 6. Separating tissue or other waste from food to support its reuse, for example as animal feed. |  |  |  |  |
| 7. Disposing of excess food in designated bins to reduce the amount of waste sent to landfills. |  |  |  |  |

**Section 10.**

**Perceived Barriers to Food Waste Reduction**

| **Barrier** |
| --- |
| The food served does not taste good or is of low quality. |
| Lack of knowledge on how to appropriately plan the quantity of food to purchase. |
| No options available for selecting portion sizes according to individual needs. |
| Reluctance to request food vendors to reduce the amount of rice or food served. |
| Insufficient information from the canteen regarding food waste reduction initiatives. |
| Lack of motivation to reduce food waste. |
| Other reasons, such as perceiving no benefits in reducing food waste. |

**Perceived Barriers to Food Waste Segregation**

| **Barrier** |
| --- |
| Time constraints during peak hours. |
| Lack of awareness regarding proper methods for separating food waste. |
| Absence of designated food waste bins in the canteen. |
| Inconvenient access to food waste bins. |
| Perception that failing to dispose of food waste in designated bins carried no personal consequences. |
| Unwillingness to separate food waste from plates due to its unpleasant or dirty nature. |
| Other reasons, such as reluctance to handle waste. |
