# Supplementary material for: Factors influencing food waste reduction in University Canteens: Toward sustainable campus waste management
Source: PLoS One. 2026 Feb 23;21(2):e0343534. doi: 10.1371/journal.pone.0343534 (PMC12928407; doi:10.1371/journal.pone.0343534)
Supplement: S1 Table — (DOCX) [file pone.0343534.s002.docx]

**S1 Table. Demographic characteristics (n=400)**

| **Demographic** | **Category** | **Number** | **%** |
| --- | --- | --- | --- |
| **Gender** | Male | 81 | 20.20 |
|  | Female | 319 | 79.80 |
| **Age** | Less than or equal to 20 years (minimum 18 years) | 310 | 77.50 |
|  | 21 years and older (up to 24 years) | 90 | 22.50 |
| **Monthly income** | Less than 15,000 baht (< $424) | 85 | 21.25 |
|  | 15,001 – 30,000 baht ($424-$848) | 87 | 21.75 |
|  | 30,001 – 45,000 baht ($849-$1,272) | 79 | 19.75 |
|  | 45,001 – 60,000 baht ($1,273-$1,697) | 55 | 13.75 |
|  | 60,001 – 75,000 baht ($1,698-$2,121) | 33 | 8.25 |
|  | More than 75,000 baht (> $2,121) | 61 | 15.25 |
