# Supplementary material for: Factors influencing food waste reduction in University Canteens: Toward sustainable campus waste management
Source: PLoS One. 2026 Feb 23;21(2):e0343534. doi: 10.1371/journal.pone.0343534 (PMC12928407; doi:10.1371/journal.pone.0343534)
Supplement: S2 File — (DOCX) [file pone.0343534.s003.docx]

**Table 1 Knowledge of food waste reduction (n=400)**

| **Knowledge question** | **True (*n*, %)** | **False (*n*, %)** |
| --- | --- | --- |
| 1. Purchasing food served by vendors without prior planning, leading to leftover waste | 349 (87.25) | 51 (12.75) |
| 2. Leaving food unfinished or uneaten, contributing to global warming | 385 (96.25) | 15 (3.75) |
| 3. Polluting wastewater from food decomposition does not cause soil and water pollution | 314 (78.50) | 86 (21.50) |
| 4. Purchasing appropriate quantities of food could reduce waste | 399 (99.75) | 1 (0.25) |
| 5. Recognizing the role that food scraps used as animal feed could play in minimizing canteen waste | 298 (74.50) | 102 (25.50) |
| 6. Feeding animals with food scraps can causes minimizing food waste canteen | 394 (98.50) | 6 (1.50) |
| Cronbach alpha = .721 |  |  |

**Table 2 Attitudes toward food waste reduction in the university canteens (n=400)**

| **Attitude** | Agree | Not sure | Disagree | **(**$\bar{\boldsymbol{x}}$**)** | **Interpretation** |
| --- | --- | --- | --- | --- | --- |
| 1. Everyone should take responsibility for their own food waste. | 382  (95.50) | 14  (3.50) | 4  (1.00) | 2.95 | High |
| 2. Leftover or uneaten food decomposes easily and does not harm the environment. | 13  (3.25) | 106  (26.50) | 281  (70.25) | 2.67 | High |
| 3. It is unnecessary for people in society to be aware of food waste impacts because other environmental problems are more serious. | 19  (4.75) | 22  (5.50) | 359  (89.75) | 2.85 | High |
| 4. Sorting food waste in canteens is complicated and time-consuming. | 20  (5.00) | 53  (13.25) | 327  (81.75) | 2.77 | High |
| 5. Sorting food waste in canteens should be the responsibility of canteen staff only. | 14  (3.50) | 41  (10.25) | 345  (86.25) | 2.83 | High |
| Cronbach alpha = .770 |  |  |  |  |  |
| **Total** | | | | **2.81** | **High** |

**Table 3 Motivation to reduce food waste in the university canteens (n=400)**

| **Motivation** | **Agree** | **Not sure** | **Disagree** | **(**$\bar{\boldsymbol{x}}$**)** | **Interpretation** |
| --- | --- | --- | --- | --- | --- |
| 1. I purchase an appropriate amount of food in the canteen, avoiding excess, to save unnecessary food expenses. | 357  (89.25) | 32  (8.00) | 11  (2.75) | 2.87 | High |
| 2. I reduce food waste in the canteen because I feel it helps conserve the resources used to produce food globally. | 335  (83.75) | 61  (15.25) | 4  (1.00) | 2.83 | High |
| 3. I separate food waste into designated bins because I feel it helps reduce pollution and protect the environment. | 361  (90.25) | 37  (9.25) | 2  (0.50) | 2.90 | High |
| 4. I am willing to follow clear canteen rules and use designated bins for food waste separation sincerely if such regulations and facilities are provided. | 383  (95.75) | 17  (4.25) | 0  (0.00) | 2.96 | High |
| Cronbach alpha = .787 |  | | |  |  |
| **Total** | | | | **2.88** | **High** |

**Table 4 Perceived behavioral control regarding food waste reduction (n=400)**

| **Perceived behavioral control** | **High** | **Moderate** | **Low** | **(**$\bar{\boldsymbol{x}}$**)** | **Interpretation** |
| --- | --- | --- | --- | --- | --- |
| 1. I am confident that I can reduce food waste when using the canteen. | 242  (60.50) | 157  (39.25) | 1  (0.25) | 2.60 | HIgh |
| 2. I can order food in quantities that match my consumption needs. | 248  (62.00) | 142  (35.50) | 10  (2.50) | 2.60 | High |
| 3. I am confident that I can properly separate food waste into designated bins. | 282  (70.50) | 116  (29.00) | 2  (0.50) | 2.71 | High |
| Cronbach alpha = .765 |  |  |  |  |  |
| **Total** | | | | **2.63** | **High** |

**Table 5 Behavioral intention in order to reduce food waste (n=400)**

| **Behavioral intention** | **High** | **Moderate** | **Low** | **(**$\bar{\boldsymbol{x}}$**)** | **Interpretation** |
| --- | --- | --- | --- | --- | --- |
| 1. I intend to reduce food waste when using the canteen. | 327  (81.75) | 71  (17.75) | 2  (0.50) | 2.81 | HIgh |
| 2. I intend to order food in quantities that match my consumption needs. | 338  (84.50) | 59  (14.75) | 3  (0.75) | 2.84 | High |
| 3. I intend to separate food waste into designated bins properly. | 362  (90.50) | 37  (9.25) | 1  (0.25) | 2.90 | High |
| Cronbach alpha = .806 |  |  |  |  |  |
| **Total** | | | | **2.85** | **High** |

**Table 6 Social influence on food waste reduction (n=400)**

| **Social influence** | **High** | **Moderate** | **Low** | **(**$\bar{\boldsymbol{x}}$**)** | **Interpretation** |
| --- | --- | --- | --- | --- | --- |
| 1. Society views separating food waste into designated bins as a socially responsible behavior. | 312  (78.00) | 84  (21.00) | 4  (1.00) | 2.77 | HIgh |
| 2. People around me, including friends and colleagues, support and encourage me to reduce food waste in the canteen. | 260  (65.00) | 126  (31.50) | 14  (3.50) | 2.62 | High |
| 3. The university’s efforts to promote food waste reduction support customers’ food waste reduction behaviors | 331  (82.80) | 68  (17.00) | 1  (0.20) | 2.83 | High |
| Cronbach alpha = .845 |  |  |  |  |  |
| **Total** | | | | **2.73** | **High** |

**Table 7 Contextual factors related to food waste reduction (n=400)**

| **Contextual factors** | **High** | **Moderate** | **Low** | **(**$\bar{\boldsymbol{x}}$**)** | **Interpretation** |
| --- | --- | --- | --- | --- | --- |
| 1. Having signs that indicate appropriate food portions helps reduce food waste. | 308  (77.00) | 73  (18.25) | 19  (4.75) | 2.72 | HIgh |
| 2.Having designated bins for food waste helps facilitate food waste separation. | 371  (92.75) | 27  (6.75) | 2  (0.50) | 2.92 | High |
| 3. Having directional signs to food waste separation points supports food waste separation. | 354  (88.50) | 41  (10.25) | 5  (1.25) | 2.87 | High |
| Cronbach alpha = .783 |  |  |  |  |  |
| **Total** | | | | **2.84** | **High** |
